# The paediatric story of human papillomavirus (Review)

**DOI:** 10.3892/ol.2014.2226

**Published:** 2014-06-04

**Authors:** IOANNIS N. MAMMAS, GEORGE SOURVINOS, DEMETRIOS A. SPANDIDOS

**Affiliations:** Department of Clinical Virology, School of Medicine, University of Crete, Heraklion 71003, Greece

**Keywords:** human papillomavirus, children, lesions, transmission, recurrent respiratory papillomatosis

## Abstract

Human papillomavirus (HPV) is composed of a particularly heterogeneous family of DNA viruses, which has gained much attention in recent years due to the discoveries of Professor Harald zur Hausen, who first identified a connection between HPV and cervical cancer. Professor Harald zur Hausen, the ‘Father of HPV Virology’, was the recipient of the 2008 Nobel Prize. HPV can be transmitted through physical contact via autoinoculation or fomites, sexual contact, as well as vertically from the HPV-positive mother to her newborn, causing subclinical or clinical infections. In infancy and childhood, HPV-associated clinical infections include skin warts, genital warts and juvenile recurrent respiratory papillomatosis, while cervical squamous intraepithelial lesions have also been reported among adolescent girls. To date, several research teams, worldwide, have extensively investigated HPV from the paediatric point of view. This primitive effort has been performed before the recent great expansion of paediatric HPV research due to the vaccination programmes against HPV, which were introduced into clinical practice in 2006. In this review article, we present a brief overview of paediatric HPV research after the first report in 1978 involving children in the research of HPV until the time point of this great expansion. In the future, it is expected that further unresolved issues will be addressed and clarified, as the paediatric story of HPV remains a challenging research target.

## 1. Introduction

Human papillomavirus (HPV), the most extensively studied virus of the past decade, is composed of a particularly heterogeneous family of DNA viruses, which has the ability to infect keratinocytes of the human skin and mucosa (1). HPV, which appears to invade the basal layer of epithelial cells, is a common pathogen associated with a wide range of cutaneous and mucosal infections (2). HPV can be transmitted through physical contact via autoinoculation or fomites, sexual contact, as well as vertically from the HPV-positive mother to her newborn and can cause subclinical or clinical infections (1,2). HPV-associated clinical infections include skin warts, genital warts, recurrent respiratory papillomatosis (RRP), low-grade and high-grade squamous intraepithelial lesions (SILs) and cervical cancer, which globally represents the second most frequent cancer in females (3).

**Figure 1:**
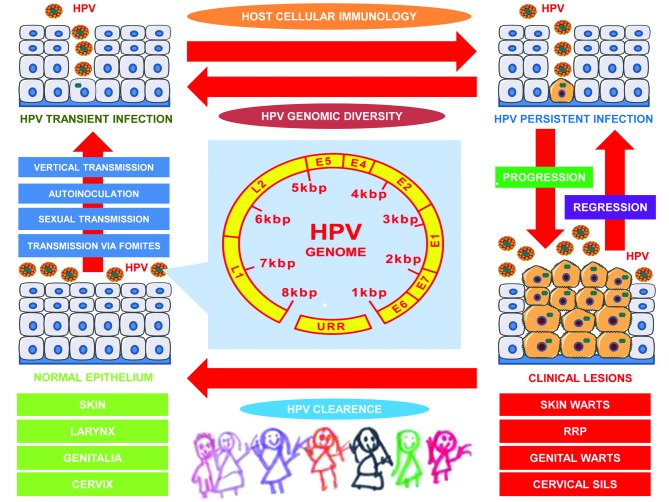
Association between HPV and clinical lesions in neonates and children. HPV can be transmitted via autoinoculation or via fomites, sexual contact or vertically from the HPV-positive mother to her newborn, initially causing HPV transient infection, which can consequently progress to HPV persistent infection. HPV persistence can either regress, or can become symptomatic, establishing clinical lesions in different anatomical sites. In infancy and childhood, HPV clearance can occur automatically. HPV, human papilloma virus; RRV, recurrent respiratory papillomatosis; SILs, squamous intraepithelial lesions; URR, upstream regulatory region.

In infancy and childhood, HPV infection involving skin warts, genital warts and juvenile RRP among both male and female neonates and children, as well as cervical SILs among adolescent girls (Fig. 1), has been excessively investigated [see reviews by Mammas *et al* (2) and Syrjänen (4)]. This scientific effort began in 1978, almost 35 years ago, when the first report involving children in HPV research was published by Pfister and zur Hausen (5). To date, several researchers, worldwide, have studied HPV from the paediatric point of view, expanding the usage of molecular techniques, such as the polymerase chain reaction (PCR) in samples obtained from children. During the past years, a great expansion has taken place in the field due to the introduction of the vaccination programmes against HPV into clinical practice. In this review, we briefly summarize some of the historical aspects of peadiatric HPV research until the time point of this great expansion.

## 2. Historical background

HPV-associated lesions, including skin and genital warts, have been reported since ancient times (6). In the 4th century B.C. Hippokrates the Asclepiad, first described skin warts, genital warts and cervical neoplasia (6–8). Although Hippokrates was certainly not the first to discover cervical neoplasia, he referred to a cervical lesion, which in Greek is termed ‘ἔλκoς’ (6), meaning ‘ulcer’ that can potentially progress to cervical cancer, indicating that HPV-associated SILs can progress to invasive cervical cancer. This knowledge referring to the physical history of HPV infection in the cervix is apparent in the impressive description by Hippokrates: ‘ɛἰ δὲ μὴ ἐμɛλɛδάνθη, μηδὲ oἱἡ κάθαϱσις ἐϱϱάγη αὐτόματη, τὸἓλκoς μέζoν ἐπoίησεν καὶ μὴ ἀνει】σα ἑκινδὸνɛυσɛν ɛἰς τὸ καϱϰινωθη̑ναι τὰ ἓλκɛα’ (7), meaning that ‘if it (the infection) is not taken care of, catharsis will not take place automatically, and thus the ulcer will increase in size and if it does not regress, there is a risk of the ulcers becoming cancerous’.

Despite the fact that skin and genital warts have been considered infectious since this early period, the development of cervical cancer due to infection was only suspected in the 19th century A.C. by an Italian scientist from Asiago, Italy, the surgeon Antonio Domenico Rigoni-Stern (9). In 1928, a Greek scientist originating from the island of Euboea, Dr George N. Papanicolaou [a brief referral to his life is presented in the article by Mammas and Spandidos (10)] observed precancerous HPV-associated lesions in vaginal smears collected from females, an observation which led to the development of the Pap smear test (11,12). The first description of HPV was provided in 1949 by Strauss *et al* (13)*,* who used electron microscopy to examine aqueous extracts of wart tissues, while in 1963 the physical properties of HPV DNA were described in the study by Crawford and Crawford (14). It was not until the 1970s, that a role of HPV in cervical cancer was postulated for the first time by Professor Harald zur Hausen, the ‘Father of HPV Virology’ (3). It is currently well established that HPV is involved in human carcinogenesis, causing not only the vast majority of cervical, but also a substantial proportion of other non-genital cancers (15).

## 3. HPV in children: a brief overview

Although the infectious cause of warts in children was known by the end of the 19th century (16), initial studies on children using molecular hybridization techniques were performed in the end of the 1970s. In an early study by Pfister and zur Hausen (5) in 1978, it was well documented that HPV 1, HPV 2 and HPV 3 predominate in skin warts in children between the ages of 5 and 15 years, while HPV 4 is most often isolated in children of older ages. As is presented in Table I, this article was the first in the literature (5,17–50) involving samples obtained from children in HPV research. Evidence of the presence of HPV in juvenile RRP also dates back to the beginning of the 1980s (17–19). These studies have provided strong evidence of the etiology of tumors caused by HPV that was verified by subsequent studies on RRP.

During the second half of the 1980s, a clear picture of the presence of specific types of HPV in genital warts in children was obtained (21,22,24). These initial reports enthusiastically supported HPV typing as an important prognostic tool for HPV-infected children, particularly in those infected with HPV 16 and HPV 18, due to the highly oncogenic potential of these two HPV types (24). For this reason, at that time, several paediatric departments were requesting HPV typing in cases with genital warts in order to identify children who were at a risk of developing cancer. At the same time, researchers evaluated the impact of the presence of HPV in the diagnosis of sexual abuse. However, early enough, it was made clear that HPV typing using molecular techniques is not sufficient to determine the source of HPV infection and pursue the possibility of sexual abuse (24,26,27). In the study by Padel *et al* (27), it was well established that HPV typing does not provide substantial evidence of the presence or absence of sexual transmission.

The presence of HPV DNA in asymptomatic neonates was initially documented in foreskins by Roman and Fife (20). Soon after the report in 1988 by Steinberg (23) addressing the transmission of HPV to the fetus, a number of studies investigated the perinatal modes of HPV transmission in childhood (25,29). These studies supported a vertical transmission mechanism of HPV in children based on the presence of HPV DNA in asymptomatic neonates in oral and genital samples at or shortly after birth (25). The detection of HPV DNA in the amniotic fluid also suggested an *in utero* mechanism of HPV transmission (25). Smith *et al* provided evidence indicating the prevalence of HPV among pregnant women increases with the gestational age from 8.0% in the first trimester to 23.1% in the third trimester (29), while, in 1994, Kaye *et al* (32) suggested that viral load is a determinant for HPV transmission to the neonate. In the study by Fredericks *et al* (30) in 1993, it was well established that the contamination of neonates occurs commonly at birth and persists for at least 6 weeks. In a subsequent report in 1995 by Cason *et al* (33), the authors supported a bimodal distribution of IgM seropositivity, which peaked between 2 and 5, and 13 and 16 years of age, suggesting that two distinct modes of transmission may occur. Perinatal HPV in infants has also been shown to be related to the mode of delivery and it was suggested that neonates are at a higher risk of exposure to HPV after vaginal delivery than after caesarean delivery (35).

In 1998, a research team from the University of Turku School of Medicine in Finland, initiated the Finnish Family HPV Study, which was the first prospective attempt to assess HPV dynamics at multiple anatomical sites in parents and infants (37). The large number of mother-infant pairs analyzed made it possible to explore the consequences of the presence of HPV in the placenta, umbilical cord blood and breast milk. Studies supporting perinatal HPV transmission have been reviewed by two separate research teams, one at the Department of Virology at Kings College in London, UK (51) and the other at the University of Turku, Finland (52). However, these reports (51,52) have been met with skepticism as regards definitive interpretation. Nevertheless, the potential impact of early acquired HPV neonatal infection on the efficacy of current vaccines for HPV-positive children remains undetermined.

The early findings by Jenison *et al* (28) in the 1990s that HPV types exist in the oral cavity of asymptomatic children were re-evaluated a decade later. In 2000, Rice *et al* (36) reported the presence of HPV in oral samples obtained from healthy children, while other researchers documented tonsillar tissue as a reservoir of HPV DNA (38–40). These findings attracted the attention of Reuters Health, raising questions concerning the modes of HPV transmission in childhood (53). Moreover, the presence of HPV in the lower tract in children may be involved in the recent increasing scientific interest of the role of HPV in lung carcinogenesis (47). Despite the detection of HPV DNA in human breast samples (41), it was clarified that this event is rare and there is no contraindication of HPV-positive mothers to breast feed their children (43,44,48,49).

In the following years, our research led out to the detection of novel HPV types, including HPV 13, HPV 39, HPV 40 HPV 56, in juvenile RRP (45). Two more studies (46,50) evaluating HPV infection in relation to neonatal prematurity and the mode of delivery remain unique in the field of pediatric HPV research. The first of these studies (46) did not find any significant evidence that maternal HPV infection is related to neonatal prematurity, while the other study (50) suggested that a caesarean section does not decrease the risk for oral HPV persistence in children. In a recent study, we used for the first time the term ‘Trojan horse oncogenic strategy’ to describe the physical history of HPV in childhood (54). This hypothesis that children act as a reservoir of silent high risk HPV types, analogous to the Trojan horse in Greek mythology, requires further investigation.

## 4. Future perspectives

Following the approval of the two current vaccines against HPV (55,56), a great expansion of studies involving HPV research and children was observed. These studies aimed to clarify several unresolved issues involving the efficacy and safety of the vaccination programmes against HPV (57–60). Moreover, they attempted to provide evidence to resolve the issues of whether or not the current target ages should be changed, and to determine the necessity of including boys into the vaccination programmes against HPV (59,60). Epidemiological studies aim to determine the factors that influence the participation of adolescents into the vaccination programmes against HPV and propose scheduled strategies to increase this participation. As the vaccination period has already begun, a re-evaluation of the potential modes of HPV transmission in infancy and the physical history of HPV-associated infections in childhood is expected. In the future, further research is required to fully investigate and clarify all these issues, highlighting the fact that the paediatric story of HPV remains a challenging research target for the next generation of researchers. Indeed, the war against HPV continues.

## Figures and Tables

**Table I:** HPV pre-vaccination research and children: a brief overview.

Year of publication	Authors/(Refs.)	Contribution
1978	Pfister and zur Hausen (5)	HPV types in skin warts in children
1981	Costa *et al* (17)	HPV types in juvenile RRP
1982	Braun *et al* (18)	HPV types in juvenile RRP
	Mounts *et al* (19)	HPV types in juvenile RRP
1986	Roman and Fife (20)	HPV types in foreskin in neonates
	Rock *et al* (21)	HPV types in genital warts in children
1987	Vallejos *et al* (22)	HPV types in genital warts in children
1988	Steinberg (23)	HPV types in genital warts in children
1989	Hanson *et al* (24)	HPV mother-to-infant transmission
	Sedlacek *et al* (25)	HPV mother-to-infant transmission
1990	Gibson *et al* (26)	HPV types in genital warts in children
	Padel *et al* (27)	HPV types in genital warts in children
	Jenison *et al* (28)	HPV types in oral samples in asymptomatic children
1991	Smith *et al* (29)	HPV mother-to-infant transmission
1993	Fredericks *et al* (30)	HPV mother-to-infant transmission
1994	Pakarian *et al* (31)	HPV mother-to-infant transmission
	Kaye *et al* (32)	HPV viral load as a determinant for mother-to-infant transmission
1995	Cason *et al* (33)	HPV mother-to-infant transmission
1996	Alberico *et al* (34)	HPV mother-to-infant transmission
1998	Tseng *et al* (35)	Evaluation of HPV infection and mode of delivery
2000	Rice *et al* (36)	HPV types in oral samples in asymptomatic children
2005	Rintala *et al* (37)	HPV mother-to-infant transmission
	Chen *et al* (38)	HPV types in tonsils in asymptomatic children
2006	Sisk *et al* (39)	HPV types in tonsils in asymptomatic children
	Mammas *et al* (40)	HPV types in tonsils in asymptomatic children
2008	Sarkola *et al* (41)	Evaluation of HPV types in breast milk
	Mammas *et al* (42)	HPV types in skin warts in children
2009	Cazzaniga *et al* (43)	Evaluation of HPV types in breast milk
2010	Mammas *et al* (44)	HPV mother-to-infant transmission
	Mammas *et al* (45)	Novel HPV types in juvenile RRP
	Mammas *et al* (46)	Evaluation of HPV infection and neonatal prematurity
2011	Mammas *et al* (47)	HPV types in lower respiratory tract in children
	Yoshida *et al* (48)	Evaluation of HPV types in breast milk
	Mammas *et al* (49)	Evaluation of HPV types in breast milk
2012	Mammas *et al* (50)	Evaluation of HPV persistence and mode of delivery

HPV, human papillomavirus; RRP, recurrent respiratory papillomatosis.

## Abbreviations

HPV: human papillomavirus
PCR: polymerase chain reaction
RRP: recurrent respiratory papillomatosis
SILs: squamous intraepithelial lesions
URR: upstream regulatory region
